# Repruducibility of tronzo and ao/asif classifications for transtrochanteric fractures

**DOI:** 10.1590/1413-78522014220500884

**Published:** 2014

**Authors:** Christian Behrendt, Thiago Batista Faleiro, Renata Da Silva Schulz, Bianca Ortiz Da Silva, Erivaldo Queiróz De Paula

**Affiliations:** 1 Hospital Naval Marcílio Dias, Rio de Janeiro, RJ, Brazil, Hospital Naval Marcílio Dias, Rio de Janeiro, RJ, Brazil; 2 Hospital Naval de Salvador, Salvador, BA, Brazil, Hospital Naval de Salvador, Salvador, BA, Brazil; 3 Faculdade Unijorge, Salvador, BA, Brazil, Faculdade Unijorge, Salvador, BA, Brazil; 4 Policlínica Naval de Niterói, Niterói, RJ, Brazil, Policlínica Naval de Niterói, Niterói, RJ, Brazil

**Keywords:** Hip fractures, Femoral fractures, Orthopedic procedures, Reproducibility of results

## Abstract

**Objective:**

To assess the reproducibility of Tronzo and AO/ASIF classifications for transtrochanteric fractures, in order to determine the most appropriate classification for clinical application, and to evaluate the influence of the level of experience of the observers in the agreement between evaluations.

**Methods:**

We selected 30 radiographic images of transtrochanteric fractures of the femur, which were presented to two groups of observers, one formed by expert physicians and the other by resident physicians.

**Results:**

When evaluated together, Tronzo classification obtained a Kappa value of 0.44. The same classification assessed by the expert group obtained a value of 0.46, while the group of residents' value was 0.44. Evaluating the AO/ASIF classification of the complete pool analysis the value found was 0.42. For the same classification, analyzed by the expert group, obtained a value of 0.41, and by the group of residents, the Kappa value achieved was 0.42. However, when analyzed in its simplified form, the AO/ASIF classification obtained Kappa values of 0.70 (pooled analysis), 0.68 (experts) and 0.72 (residents), considered concurrent.

**Conclusion:**

The AO/ASIF simplified classification showed substantial reproducibility and is, therefore, recommended as the most suitable for clinical application. The level of experience of the observers did not influence significantly the agreement between evaluations. *Level of Evidence III, Diagnostic Study - Investigating a Diagnostic Test.*

## INTRODUCTION

Classification systems in the areas of Orthopedics and Traumatology were introduced a long time ago and it is believed to have preceded the advent of radiography, after which these systems gained even more importance. The use of ratings plays a crucial role in defining the severity, treatment and prognosis of the fracture, as well as ensuring a more accurate comparison of results and facilitating the establishment of guidelines for evaluation and choice of treatment.^1^

The classifications of fractures should be simple, easy to remember so that they are universally applicable and acceptable and have much influence on the choice of treatment as in the definition of prognosis.^2^^,^ 3 A key prerequisite for a classification proposal is its ability to be reproduced among observers. The purpose of the ratings is, therefore, to assist the surgeon to choose an appropriate method of treatment, as well as to provide a reasonably accurate estimate of the results to be obtained.^4^

The agreement between observers (inter-observer) is of paramount importance for its adequate use, both in clinical practice and in scientific research. In this context, the use of simple classifications aims to minimize the lack of uniformity of the parameters used in the clinical evaluation, determining prediction in terms of prognosis of the injuries.^2^

The trochanteric fractures are fractures of the proximal end of the femur, extracapsular, whose trace lies between the greater and lesser trochanters. In the USA, the annual proportion of trochanteric fractures in the elderly is approximately 63 fractures per 100,000 individuals and among men, this proportion drops to 34 per 100,000 indivíduos.^5^ About 90% of these types of fractures in the elderly result from a simple fall.

The assessment according to the stability of the fracture is important for surgical planning. Some factors such as bone quality, type of fracture line, quality of reduction achieved, type of implant used and placement of the implant in the bone directly influence the assessment.^6^ Classification systems have been proposed by some authors aimed to guide therapeutic decision and observation of stability.

There are various classification systems described for trochanteric fractures, considering mainly the deviation and the presumed stability after reduction. The most commonly used systems are the classifications of Tronzo; Evans; Boyd-Griffin and the classification system AO/ASIF.^6^^-^ 11

According to the Tronzo classification system, a single trait fracture without deviation is classified as type I. A simple line fracture, with fragments deviation is a type II; type III are fractures in which there is deviation and comminution of the posteromedial wall with the tip of the calcar impacted into the medullary canal of the distal fragment, the latter being medialized; in the type III variant, the greater trochanter is also fractured; type IV comprises fractures with deviation and posteromedial comminution, in which the distal fragment is lateralized and the proximal fragment is medialized with the tip of calcar outside the medullary canal; in type V the fracture presents with reverse obliquity, i.e. fracture with inverted trace of medial proximal to lateral-distal. Fractures of types I and II are considered stable while the unstable are the types III, variant, IV and V. Instability criteria of trochanteric fractures included reverse obliquity, posteromedial comminution and sub-trochanteric extending.^8^

Recently, there was an increase of adherents to the Müller's system of the Association for the Study of Osteosynthesis/Association for the Study of Internal Fixation (AO/ASIF), who organized the fractures of long bones in hierarchical triads, according to severity. This system established a record that should be the basis for treatment and evaluation of results.^9^ According to this alphanumeric classification system, trochanteric fractures are type 31A. These fractures are divided into three groups and each group in three subgroups. Fractures of the first group are simple fractures (in 2 parts) with a single extension into the medial cortex, and the lateral cortex of the greater trochanter remains intact. Subgroups further define the geometry of the fracture line. The subgroup 1.1 presents with fracture line on intertrochanteric line; in subgroup 1.2 the fracture line passes through the greater trochanter; in subgroup 1.3, below the lesser trochanter. Fractures in group 2 are multi-fragmented; the fracture line may start at any region of the greater trochanter, extending medially into two or more directions. This creates a third fracture fragment, which includes the lesser trochanter. The lateral cortex is intact and the subgroups define the number and geometry of the fragments, subgroup 2.1 which presents with an intermediate fragment; subgroup 2.2 shows with several intermediate fragments; and subgroup 2.3 present fractures comprising those with greater than 1 cm beyond the small trochanter extension. Moreover, fractures in group 3 are those with both cortices fractured, medial and lateral, with reverse obliquity, and its subgroups describe the direction and fracture comminution. In subgroup 3.1, the trace is simple oblique; in the subgroup 3.2, simple transverse; and in 3.3 in the fracture is multifragmentar.^12^^,^ 13

The objective of this study is to evaluate and compare the reproducibility of Tronzo and AO/ASIF classifications for trochanteric fractures, using the *Kappa* coefficient (Κ). Despite the frequency with which the trochanteric fractures occur, there are no studies in the literature that indicate which of the two most widely used systems in our midst (Tronzo and AO/ASIF) is more reproducible. As a secondary objective of the present study, we seek to evaluate the influence of the level of experience of the observers in the concordance of classification systems studied.

## MATERIALS AND METHODS

This study was performed at the of Traumatology and Orthopedics ward in a tertiary military hospital, being characterized as descriptive and qualitative exploratory. It has been submitted to and approved by the Research Ethics Committee of the institution.

There were randomly and retrospectively selected 30 unidentified patients' radiographs showing trochanteric fractures of the femur, from the radiographic file of the researchers team.

The radiographs were photographed and the images placed into a digital file, along with illustrations and detailed explanations of Tronzo and AO/ASIF classification systems, in order to minimize bias due to difficulties in interpreting the classification and/or any possible oblivion.

The radiographs were presented to two groups of observers, based on level of experience, always in the same order, without time limit for the classification of fractures. Group A consisted of ten clinical medical assistants with a specialist degree by the Brazilian Association of Orthopedics and Traumatology, and group B, ten resident doctors in Trauma and Orthopedics.

To evaluate the inter-observer reproducibility, the *Kappa* index (Κ) stratified by Landis and Koch was used.^14^ The sample size calculation was performed according to statistical parameters and based on previous studies.

The *Kappa* coefficient can vary from -1 (complete disagreement) to +1 (complete agreement). There is no precise definition of acceptable levels of agreement. The most used concordances and reproducibility values are subdivided into three types: poor (0 to 0.5), good (0.51 to 0.75) and excellent (0.76 to 1).^15^

Landis and Koch^12^ classified as poor (below 0), mild (0-0.2), low (0.21 to 0.4), moderate (0.41 to 0.6), substantial (0.61 -0.8), and almost perfect (0.81 to 1).

## RESULTS

The analysis of inter-observer agreement of Tronzo and AO/ASIF classification was built by the *Kappa* index, obtained after classification of 30 radiographs by 20 observers, together and divided into two groups (experts of the Brazilian Society of Orthopedics and Traumatology and Residents), considering that the image quality was satisfactory for the classification task.

For the Tronzo classification system, analyzed by the two groups, a *Kappa* value of 0.44 was obtained, considered of moderate reproducibility. For the same classification, analyzed by the expert group, we obtained a value of 0.46, while the group of residents, the value was 0.44, both also considered of moderate reproducibility.

Regarding the complete AO/ASIF classification, including its nine subtypes a value of 0.42 was obtained, when analyzed together by both groups. When analyzed separately by the group of experts, the value found was 0.41, while for the group of residents was 0.42, all considered of moderate reproducibility. For the same classification, in the incomplete modality (with only 3 subtypes), the Kappa value in the joint analysis by the two groups was 0.70. In the analysis by the group of experts *Kappa* was 0.68, while for the group of residents, a kappa value of 0.72 was obtained. Thus, for the simplified classification used, the *Kappa* values are in a substantial agreement range.

## DISCUSSION

The inter-observer match between the classification systems of several fractures in Trauma- Orthopedics has been the research subject of several authors. Congalton^16^ reports that the use of the *Kappa* coefficient is satisfactory in assessing the accuracy of a thematic classification, since it takes into account all the complex aspects in its calculation, including elements outside the main diagonal, which represent the disagreements in the classification unlike the overall accuracy.

Recent studies related to the inter-observer reproducibility of the Tile classification for acetabulum fractures and the Schatzker classification for tibial plateau fractures, showed moderate level of inter-observer agreement on classification, also pointing out the lack of statistically significant difference regarding the analysis conducted by residents and specialists.^17^^,^ 18

Beaule *et al.* 19 found substantial reproducibility (*Kappa* 0.70) for Letournel classification of acetabular fractures. Computed tomography was useful for identifying articular impaction, but does not seem essential for the classification of acetabular fractures.

Although the use of clinical evaluation ratings in Trauma and Orthopedics specialty, is a fairly widespread practice, although some studies show a poor inter-observer reproducibility, a fact that should be analyzed in order to establish alternatives for improving uniformity to allow the greatest possible reproducibility. The Garden classification for femoral neck fractures demonstrated poor inter-observer reproducibility in the study of Gusmão *et al.* 1

Wainwright *et al.*,^20^ in a study on inter and intra-observer reproducibility for distal humeral fractures, noticed that the classification of Riseborough and Radin^21^ showed moderate match, but half of the fractures could not be classified by this method. However, full AO classification showed low reproducibility (Kappa 0.343). When incomplete (only 3 subtypes), its match was moderate (Kappa 0.52).

Brady *et al*.^22^ demonstrated that the Vancouver classification for periprosthetic femoral fractures shows good reproducibility (*Kappa *0.78). However, the AO/ASIF classification, also regarding periprosthetic femoral fractures, showed low inter-observer reproducibility, when complete (*Kappa* 0.33).

Regarding trochanteric fractures of the femur, Schwartsmann *et al.*,^10^ investigating the reproducibility of the AO classification for these fractures, showed that, when complete (with nine subtypes), this classification showed unacceptable levels of reproducibility (*Kappa* 0.34). However, when simplified (with only three subtypes), it was considered good or substantial. In another survey, Pervez *et al*.^23^ evaluated 88 transtrochanteric fractures among five observers, coming to the conclusion that the AO classification with subgroups and Evans classification are not acceptable, and recommend the use of AO without subgroups. Jin *et al.* 24 also demonstrated that the AO classification, when incomplete (analyzing only A.1, A.2 and A.3 types), has a higher reproducibility than Evans'^7^ and Boyd and Griffin's^11^ systems. However, when complete, the AO classification showed poor reproducibility for these fractures.

## CONCLUSION

From the data obtained after analysis of radiographic images by the two groups involved in this research, and taking into consideration other works on classification of several fractures, including transtrochanteric, we conclude that complete AO/ASIF and Tronzo ratings show lower inter-observer reproducibility compared to the simplified AO/ASIF classification. While the former are in the moderate performance reproducibility range, the latter is in substantial performance range, and is, therefore, recommended by the authors as the most suitable system for clinical application.

Regarding the comparison between groups, we conclude that the level of experience of the observers showed no significant influence on the reproducibility of the ratings between the two groups in the present study (specialists and residents).
